# Władysław Szymonowicz (1869–1939)

**DOI:** 10.1007/s00415-024-12625-5

**Published:** 2024-08-21

**Authors:** Marta Pugaczewska, Andrzej Grzybowski

**Affiliations:** 1 Department of General, Dental and Interventional Radiology, Pomeranian Medical University, Szczecin, Poland; 2 Institute for Research in Ophthalmology, Foundation for Ophthalmology Development, Poznan, Poland; 3 Department of Ophthalmology, University of Warmia and Mazury, Olsztyn, Poland

**Keywords:** Władysław Szymonowicz, Ladislaus Szymonowicz, History of neurology

**Fig. 1:**
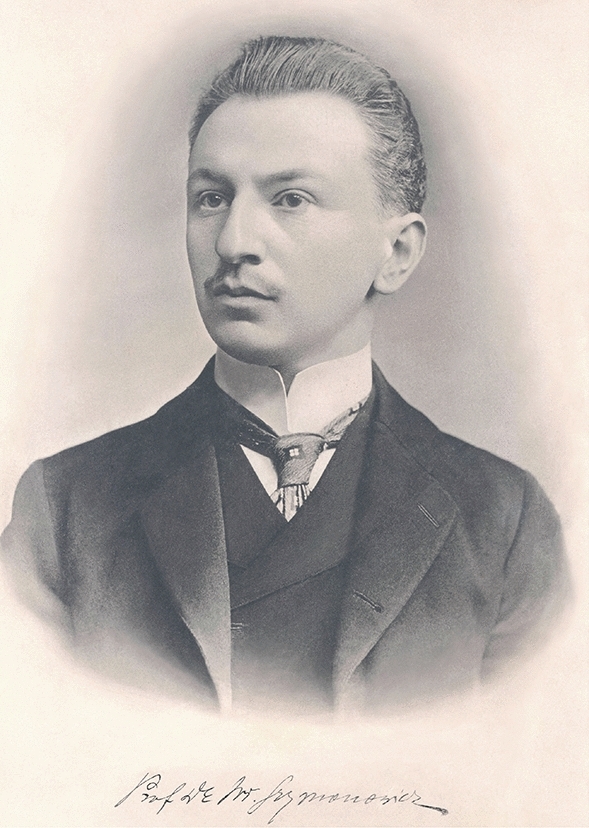
Władysław Szymonowicz (1869–1939). Source: Władysław Szymonowicz Library and Memorial Museum, Department of Histology, Cytology and Embryology at the Danylo Halytsky Lviv National Medical University

Władysław (Ladislaus) Szymonowicz (Fig. 1) was a Polish histologist and embryologist of Armenian descent, author of pioneering scientific works, in particular regarding nerve endings in the skin of humans and animals. Moreover, his strong interest in histology led Szymonowicz to complete a “Textbook of histology and microscopic anatomy” (Lehrbuch der Histologie und der mikroskopischen Anatomie mit besonderer Berücksichtigung des menschlichen Körpers einschliesslich der mikroskopischen Technik, Stuber, Würzburg, 1901), which received international recognition.

Szymonowicz was born on March 21, 1964 in Tarnopol (then in Galicia, Austria-Hungary, today Ternopil in Ukraine) into a family of jurists and clerics [1]. His father, Władysław senior, was a lawyer and nephew of Grzegorz Michał Szymonowicz (1800–1875), Archbishop of the Armenian Rite Archdiocese of Lviv. Szymonowicz junior’s mother, Joanna, née Kosińska, also came from an Armenian family. After graduating from the Gymnasium in Lviv in 1887, Władysław Szymonowicz enrolled in the Faculty of Medicine of Jagiellonian University, Kraków, where in the fourth year of studies he became an assistant in the Department of Physiology. While a student, Szymonowicz began research on the nerve endings of hair follicles, which, a year before finishing medical school, resulted in the publication of his first scientific article, on the fine morphology of nerve endings in the vibrissae of the albino mouse [2].

In March 1893, Szymonowicz received his diploma of doctor of medical science. In the same year, under the supervision of the departmental head, Napoleon Cybulski (1854–1919), Szymonowicz studied the morphology and functions of the adrenal glands. As a result of a series of animal experiments, Szymonowicz and Cybulski formulated a hypothesis on the secretion of a substance into the bloodstream by the adrenal medulla indispensable for life, with a marked impact on several vital functions, including blood pressure, and named the substance, which had not yet been isolated, “epinephrine” or “suprarenin”. These findings became important in determining the then unknown function of the adrenal glands, and eventually helped to discover catecholamines [3]. Szymonowicz’s publication of a work on the structure and development of the adrenal glands and the results of investigation on their function earned him an MD/PhD degree in 1896 [4].

Scientific success led to a scholarship for journeys to Berlin in 1895 and 1896, where Szymonowicz collaborated with Rudolf Virchow (1821–1902) and Oscar Hertwig (1849–1922). While working with Hertwig, Szymonowicz described nerve fibers branching from Merkel touch cells in the epidermis, named them connecting nooses (*ansae*), and classified them as specifically differentiated formations, organizing distant areas of the epidermis into receptive fields of appropriate groups of touch cells. He also suggested the function of Merkel cells and their associated nerve terminals as mechanoreceptors [5]. That report made the name of Szymonowicz internationally known.

In February 1897, Szymonowicz went to the University of Lviv, where he initially served as assistant professor, and from 1898 as chair of the first independent Department of Histology and Embryology in Poland. In 1903, he was promoted to full professor and started organizing a research institute. In 1906–1907, he served as dean of the Faculty of Medicine of the University of Lviv. While in Lviv, Szymonowicz devoted much of his research effort to sensory structures. He had a rich collection of specimens, which led to the establishment of a museum with 127 wax embryological models, 120 anatomical models, and about 6,000 histological and embryological slides [6].

After the First World War, Szymonowicz continued his scientific activity, which had been temporarily interrupted by the war, in now free Poland. He owned rich collections of microscopic tactile hair preparations. Szymonowicz also improved existing and developed new preparation and staining methods. In 1926, he was the first to describe the two-meniscus form of the Merkel disc [7]. In 1927, he traveled to Utrecht and Madrid to visit the centers of prominent neurohistologists, such as Jan Boeke (1874–1956) and Nobel laureate Santiago Ramón y Cajal (1852–1934). In the following years, Szymonowicz’s works on the innervation of the mammalian nasal hairs were widely referenced. Based on the autopsy material of human fetuses and newborns collected over the preceding years, he presented details on the successive developmental stages of nerve endings in the human skin [8, 9].

An important contribution to the teaching of histology internationally was the publication in 1901 of the German-language “Textbook” mentioned earlier, which was reissued in five revised editions (in 1909, 1915, 1921, 1924 and 1930) and translated into Italian, English, Polish and Spanish, with some parts also translated into Japanese.

Szymonowicz died on March 10, 1939 in Lviv. He was interred in the family crypt at Lyczakowski Cemetery.

In the mind of his colleagues, Szymonowicz remained a gentleman who rarely taught classes, so that he could fully devote himself to research [10].
